# Supplementary material for: Effects of Nutrition Counselling and Unconditional Cash Transfer on Child Growth and Family Food Security in Internally Displaced Person Camps in Somalia—A Quasi-Experimental Study
Source: Int J Environ Res Public Health. 2022 Oct 18;19(20):13441. doi: 10.3390/ijerph192013441 (PMC9603782; doi:10.3390/ijerph192013441)
Supplement: Supplementary file 1 [file ijerph-19-13441-s001.zip › ijerph-1948873-supplementary.pdf]

Supplementary Table S1. Intervention effects on children's weight-for-height, weight-for-age and height-for-age Z scores

	Baseline	Follow-up	Absolute change in mean z score between baseline and follow-up	Adjusted coefficient (95% CI)	*p*
**WHZ, mean (SD)**					
NC	-0.73(1.08)	-0.58(1.39)	-0.15	0.20 (-0.31-0.72)	0.42
NC+UCT	-0.96(1.02)	-0.66(1.34)	-0.3	-0.35(-0.21-0.92)	0.22
Control	-0.67(1.05)	-0.73(1.29)	0.06	1	
**WAZ, mean (SD)**					
NC	-1.46(1.16)	-1.83(1.05)	0.37	-0.99(-0.53-3.27)	0.65
NC+UCT	-1.75(1.01)	-1.79(1.22)	0.04	0.26(-0.18-0.71)	0.25
Control	-1.57(1.13)	-1.87(1.10)	0.3	1	
**HAZ, mean (SD)**					
NC	-1.72(1.59)	-2.46(1.38)	0.74	-0.26(-0.74-0.22)	0.29
NC+UCT	-1.95(1.53)	-2.36(1.55)	0.41	0.04(-0.42-0.50)	0.86
Control	-1.95(1.51)	-2.35(1.44)	0.4	1	
